# Compartments and Connections Within the Germinal Center

**DOI:** 10.3389/fimmu.2021.659151

**Published:** 2021-03-31

**Authors:** Domenick E. Kennedy, Marcus R. Clark

**Affiliations:** Gwen Knapp Center for Lupus and Immunology Research, Section of Rheumatology, Department of Medicine, University of Chicago, Chicago, IL, United States

**Keywords:** B cell, germinal center, transcription, epigenetics, T cell help, tingible body macrophage, somatic hypermutation, affinity maturation

## Abstract

Protective high affinity antibody responses emerge through an orchestrated developmental process that occurs in germinal centers (GCs). While GCs have been appreciated since 1930, a wealth of recent progress provides new insights into the molecular and cellular dynamics governing humoral immunity. In this review, we highlight advances that demonstrate that fundamental GC B cell function, selection, proliferation and SHM occur within distinct cell states. The resulting new model provides new opportunities to understand the evolution of immunity in infectious, autoimmune and neoplastic diseases.

## Introduction

Since the histologic identification in 1930, almost a century of investigation has revealed the central importance of germinal centers (GCs) in humoral immunity (1). Fundamental to GC function is the orchestration of the molecular programs of immunoglobulin gene somatic hypermutation (SHM), selection for antibody affinity and specificity, and proliferative expansion of selected cells. Within the GC, these processes are coordinated with remarkable rapidity such that a B cell transits through these processes in four to six hours allowing for numerous rounds of selection and immune amplification during the course of a typical acute GC reaction of 14 to 21 days (2, 3). From the GC circuit both plasma cells (PCs) and memory B cells (MBCs) are produced ensuring both acute and durable antigen-specific immunity.

The importance of GCs in infection and vaccine responses has been demonstrated in numerous studies (3–5). However, the molecular nature of the GC incurs risk. One risk is that, through stochastic SHM, autoreactive and potentially pathogenic antibodies arise. However, elegant studies have demonstrated that GCs strongly select against autoreactivity (6). Indeed, selection against autoreactivity appears to have primacy over selection for affinity. The mechanisms by which GCs purge the antigen-selected repertoire of autoreactivity remain unclear.

The other risk of ongoing SHM and proliferation is neoplastic transformation. The GC B cell molecular program establishes a state that promotes survival in the presence of increased genomic stress (7, 8). This both enables SHM and increases the risk of lymphoma (9, 10). Indeed, GC B cells are precursors for large B cell lymphoma, follicular lymphoma, and Burkitt lymphoma (11, 12). Transformation has been linked to off-target AID activity (13). Studies in humans and mice have revealed multiple genetic, epigenetic and signaling mechanisms that individually mitigate the risk of neoplastic transformation inherit to the GC response. Recent studies are providing insights into how these mechanisms are coordinated with those that drive affinity maturation (14–16).

In this review, we will discuss the current understanding of how SHM, selection and proliferation are coordinated within the GC. We will first discuss the cellular architecture of the GC and then what is known about underlying molecular programs. Finally, we will propose a model that segregates fundamental GC molecular functions into separate cell states and niches in a way that enables effective adaptive immunity and mitigates the risks of neoplastic transformation.

## GC Cellular Evolution

The initial histological description of GCs noted characteristic dark and light zones (DZ and LZ respectively). Live cell imaging, and other complementary approaches, have provided remarkable insights into how GCs form and polarize into these two zones that perform very different functions. However, as described below, it has become clear that simple division of cells into DZ and LZ populations obscures underlying molecular GC functions.

### Germinal Center Initiation

Activation of naïve B cells in primary follicles induces migration to interfollicular areas and conjugates with antigen-specific T cells. B cells then present antigens to T cells and receive help through CD40-CD40L signals that promote B cell survival (5, 17, 18). Based on intravital microscopy experiments, these B-T cell conjugates can be found within a day after immunization (19, 20). NF-κB signaling is critical downstream of BCR activation to form GCs (21). However, while GCs do not form in mice with impaired NF-κB signaling, responding B cells are still able to migrate to the B-T cell border and present antigen to T cells. Instead, NF-κB signaling regulates expression of the transcription factors (TFs) IRF4 and BCL6, which are critical for both the GC and PC developmental programs (18, 20, 22, 23).

The formation of B-T cell conjugates is followed by T cells entering the B cell follicle on day 3. Subsequently, on day 4, B cells re-enter the B cell follicle and proliferate to form early GCs (18–20). Contrary to these distinct cellular events, the molecular regulation underlying the early events after antigen encounter is still being defined. Recent studies suggest there are multiple pre-GC B cell states leading up to commitment to the GC B cell program (24). Some of these are controlled by the transcriptional repressor BCL6 (20, 25). BCL6 is upregulated in the outer follicle after antigen encounter and is important for forming B-T cell conjugates prior to GC commitment (20). Within early and mature GCs, BCL6 controls B cell positioning within the B cell follicle (20, 23, 26–28). It also enhances GC B cell proliferation by making them more tolerant of DNA damage (7, 8). Ultimately, BCL6 drives the GC B cell program and prevents PC differentiation through inhibition of *Prdm1* (BLIMP) (29).

Recent studies further reinforce the importance of BCL6 in GC B cell commitment. By modulating the amount of Bcl6 expression in transgenic mice, one study found that Bcl6^hi^ B cells responding to immunization were more likely to commit to the GC program (30). In complementary studies, Zhang et al. found that while T-B cell conjugates are important to generate GC B cell precursors, increasing the time of T-B cell conjugates or CD40 signaling reduces progression to a Bcl6^hi^ state and favors plasmablast (PB) differentiation in the extrafollicular region (24).

### Class Switch Recombination Occurs During the GC Initiation Phase

In 2019, it was discovered that class switch recombination (CSR), the process by which B cells perform DNA rearrangements at the heavy chain locus to replace IgM and IgD, for IgA, IgG, or IgE, occurs during the GC initiation phase (31). Most studies on CSR have been performed *in vitro* and focused on the molecular mechanism, reviewed (32). However, strong evidence for when CSR occurs *in vivo* was lacking. Through a combination of imaging and molecular experiments Roco et al. found that CSR occurs in the first few days after activation and prior to GC commitment (31). Evidence for this included the observation of predominantly IgM^+^ GCs as well as the visualization of CSR prior to mature GC formation. This study resolved a critical question in the field placing CSR in the early events during GC initiation, and validated earlier evidence that CSR might occur prior to mature GCs (33, 34). The transcriptional states associated with CSR have now been resolved at the single cell level, further accelerating our understanding of early events in the GC reaction (35).

### Mature GC Cellular Dynamics

By day 4 after immunization, GCs precursors begin to expand and polarize to form LZ and DZ areas by day 7 (18, 36). The LZ contains more sparse populations of B cells that capture antigen from follicular dendritic cells (FDCs) and receive help from cognate T follicular helper (T_FH_) cells (37). B cells in the LZ are selected based on their competency to present antigen to T_FH_ cells as well as BCR signal strength (38–40). These B cell interactions with T_FH_ cells guide the major known GC fates which include cyclic re-entry, cell death as well as PC and MBC differentiation (37). Tingible body macrophages (TBMs), which lie within the DZ, clear dying B cells and thereby likely prevent inflammation and autoimmunity (37, 41).

A wealth of data assign both proliferation and somatic hypermutation to the DZ (3, 18, 37). However, genomic mutation, and the attendant genotoxic stress are incompatible and indeed antagonistic to proliferation. It is possible that mechanisms intrinsic to genotoxic stress, such as the sensing of DNA breaks by p53, segregate proliferation from SHM within the DZ (42). However, as discussed below, these incompatible processes occur in different cell populations each occupying a unique niche within the GC.

### A Paradox

An extensive body of literature has revealed that the LZ and DZ perform very different functions and that this is associated with great molecular complexity. However, when LZ (CD83^hi^CXCR4^low^) and DZ(CD83^lo^CXCR4^hi^) cells are isolated and characterized for RNA expression and genomic accessibility, they are remarkably similar (14, 38, 43). Taken at face value, these data suggest that primarily post-transcriptional mechanisms regulate cycling through the dark and light zones. This seems attractive as it could provide for very rapid cell fate transitions. Alternatively, it is possible that simply dividing GC B cells into two populations obscures important underlying molecular dynamics.

There are data supporting the latter possibility. In addition to driving affinity maturation, the GC selects for differentiation into both PCs and MBCs. Precursors of these populations must exist in the GC and, indeed, some have been identified (16, 44, 45). However, these populations are not observed upon simple division of the GC into two populations.

Given the rapidity of the GC cycle, it is also possible that B cells are always in transition (46). This is suggested by available single cell RNA-Seq studies, where GC B cells do not resolve into discrete cell populations (14–16). However, some cell fate decisions are discrete and are associated with definitive checkpoints. A cell either undergoes mitosis or it does not. Likewise for apoptosis. It is possible that SHM occurs along a gradient of cell states. However, we would argue that such a strategy would incur unnecessary genomic risk. Furthermore, during B lymphopoiesis genomic recombination and proliferation are segregated into very different cell populations (47, 48). As described below, this strategy is recapitulated in the GC.

### Division of the DZ Into Two Discrete Cell States

The canonical DZ encompasses a gradient of CXCR4 and CD83 surface expression. Differing levels of CXCR4 raises the possibility that these cells could occupy different niches within the DZ and therefore be imbued with different functions. Remarkably, when we split the DZ into two populations (CXCR4^hi^CD83^+^ and CXCR4^+^CD83^-^), and therefore GC B cells into three populations, there were striking differences in RNA and protein expression (14). It became clear that proliferation was restricted to the CXCR4^hi^CD83^+^ population. More specifically, these cells were the site of mitosis marked by increased cell size, enrichment of cells in G2/M and high expression of the mitotic factor Cyclin B1. Previous work has indicated that GC B cells can transit the G1/S checkpoint in any compartment (49, 50). Our data indicate that mitosis occurs in a discrete cell state, which we refer to as proliferative DZ cells or DZp cells.

Conversely, CXCR4^+^CD83^-^ cells have features of cells undergoing SHM. These include high expression of *Aicda* and genotoxic stress genes as well as induced phosphoproteomic pathways of DNA damage response. These cells were also in the G1 phase of the cell cycle where AID activity is known to occur (14, 51). We refer to this DZ subset as DZd for DZ differentiation. While immunoglobulin gene transcription, which is necessary for AID targeting (52), is induced in DZd, it was effectively repressed in DZp cells. These and other data indicate that both positive and repressive mechanisms ensure segregation of mitosis and SHM into different cell states.

Comparison across RNA expression and proteomic data sets indicated a cyclic progression in which cells selected in the LZ transit to the DZp for mitosis and then to the DZd for SHM (14). These data also revealed how the molecular programs in one cell state primes for functions in the next. Myc provides an example. The *Myc* locus displayed increased genome accessibility and transcription in LZ B cells compared to the DZ populations. However, phosphorylated MYC protein was observed in LZ and DZp B cells, with the downstream MYC program uniquely high in DZp cells.

These data, in conjunction with scRNA-Seq data (14, 15) indicate that expression across each stage is dynamic and that the initiation of transcriptional programs rapidly induces proteomic and functional programs as cells transit through each stage. Overall, these data help explain the rapidity of the GC cycle (2, 3, 38, 50, 53–57).

Remarkably, ATAC-Seq of the LZ, DZp and DZd revealed enhancer accessibility was also very dynamic across the three cell states (14). These differences were not only quantitative but also qualitative with characteristic TF binding motifs becoming accessible in each GC B cell state. In fact, the most enriched TF binding motifs found in each subpopulation were for TFs known to have importance within the GC. These included CTCF in the DZd, OCT2 in the DZp, and SpiB and PU.1 in the LZ (58–62). OCT2 is required for GC B cell proliferation and is dysregulated in B cell lymphomas (62). While OCT2 binding motifs were enriched in DZp accessible regions, *Oct2* expression did not change between subsets. Instead, expression of *Pou2af1* (binding partner of OCT2) was increased in DZp cells (14, 63–65). These data suggest that epigenetic mechanisms play important and complementary roles to TFs in regulating GC B cell fate. We propose that, by mechanisms yet to be defined, GC B cells integrate regulatory mechanisms across the whole vertical pathway of protein expression to affect rapid cell fate decisions.

### A New GC Model

**Figure 1:**
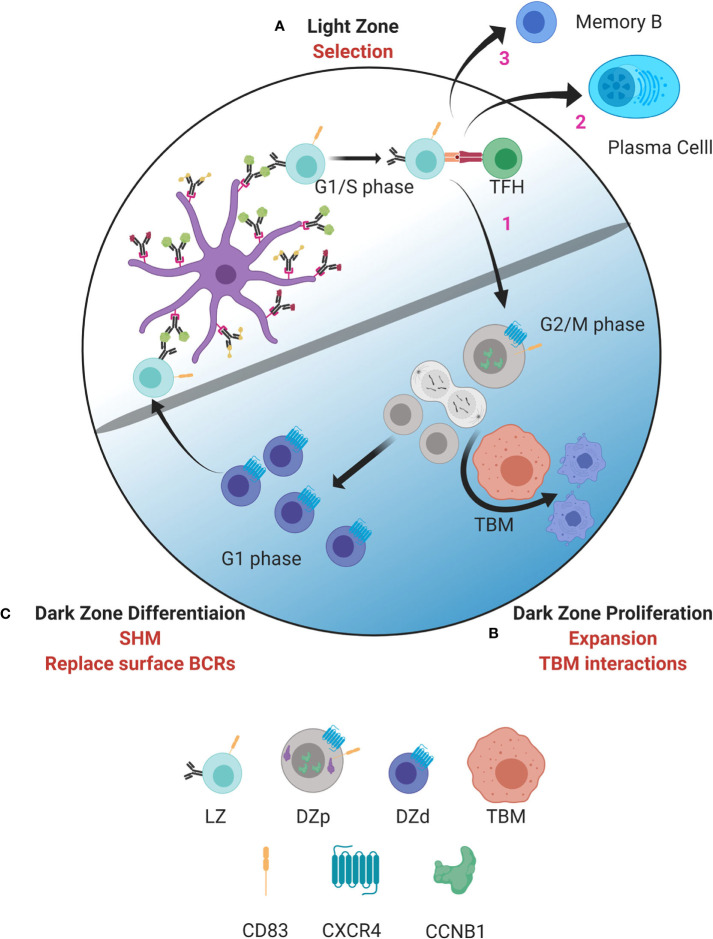
Model of germinal center dynamics and compartmentalization. GC B cells progress through a series of molecular states compartmentalizing key functions to distinct spatial niches. **(A)** GC B cell selection occurs in the LZ. B cells entering the LZ from the DZd first attempt to capture antigen deposited on follicular dendritic cells (FDC). This is followed by antigen processing and presentation to T_FH_ cells in the context of MHC class II. Interactions between LZ B cells and T_FH_ determine if B cells are fated to differentiate into MBCs or PBs, undergo cyclic re-entry into the DZp, or initiate apoptosis. Cells selected for cyclic re-entry migrate to the DZp **(B)**. We propose that B cells that initiate apoptosis in the LZ are cleared by TBMs in the DZp niche. Those cells that are successfully selected in the LZ, and therefore are not cleared by TBMs, undergo mitosis. **(C)** After one or more rounds of cell division, GC B cells transit to the DZd compartment where differentiative functions, such as SHM and replacing old BCRs with newly mutated BCRs, are proposed to occur. B cells that successfully complete processes in the DZd migrate to the LZ to undergo selection. Figure created with BioRender.com.

Our analysis revealed several markers of the DZp population that allowed identification of distinct clusters of DZp cells within the larger DZd pool (14). These clusters did not arise solely from clonal proliferative expansion. Rather, cells appear to migrate to these DZp niches to undergo mitosis. Why would mitosis occur in a distinct niche? Subsequent analysis demonstrated that DZp cells are intimately intertwined with TBMs. Furthermore, within these cellular aggregates are GC DZp cells that are apoptotic and appear to be undergoing engulfment by TBMs. Integration of these and molecular data suggests a new model of GC compartmentalization (**Figure 1**).

As well described, selection for antigen receptor affinity occurs within the LZ with coordinated collaboration between GC LZ B cells, FDCs and T_FH_ cells (**Figure 1A**). Interactions between LZ B cells and T_FH_ cells are critical to determine GC B cell fate. Our data suggest that those LZ B cells that have been successively selected, and also those that have not, migrate to the DZp niche (**Figure 1B**). Those fated for apoptosis are then eliminated by TBMs, which are the principal macrophage cell population within the GC (37, 66). The mechanisms by which TBMs identify dying B cells is not fully understood. However, it is known that FDCs secrete a molecule called MFGE8, which labels apoptotic cells for phagocytosis (67, 68). This labeling occurs by binding phosphatidylserine that is externalized on the surface of cells undergoing apoptosis (69). Recently, a molecule called decay accelerating factor (DAF) has also been proposed to regulate GC B cell phagocytosis (70). Such signals likely help TBMs distinguish between healthy B cells and B cells undergoing apoptosis. We propose that B cells that have been successfully selected in the LZ, and are not cleared by TBMs, can then undergo mitosis.

Why would clearance of apoptotic cells be coordinated with mitosis? Apoptosis is the usual pathway of GC cell death (37). However, when apoptotic cells attempt division it can result in mitotic catastrophe and necrosis (71). Therefore, by positioning TBMs within the DZp niche, necrosis, and the attendant inflammation, would be prevented. Many factors affect the G1/S checkpoint and the decision to initiate proliferation (72). Our data suggest that the main quality check for cell cycle progression is at G2/M.

One of the main controllers of proliferation within the DZp is Myc. B cells selected for cyclic re-entry typically divide on average twice in the DZ (49, 50). The number of divisions is dependent on the strength of signal received from T_FH_ cells. This, in turn, correlates with Myc protein levels implicating Myc in controlling the extent of proliferation (73). In this model, selection in the LZ determines the magnitude of Myc expression. Proliferation then continues until Myc levels are sufficiently diluted by cell division (73–75).

Upon completing one or more rounds of mitosis, GC B cells transition to the DZd stage (**Figure 1C**) where cell cycle exit is coordinated with induction of immunoglobulin gene transcription, which is required for AID targeting. Furthermore, dissolution of the nuclear membrane, as occurs when cells undergo mitosis, facilitates AID entering the nucleus (51). These mechanisms are predicted to restrict AID-mediated SHM to DZd cells. Indeed, these cells bear features of genotoxic stress and DNA repair associated with SHM (14). Our findings are consistent with studies suggesting that SHM occur in G1 phase cells (51, 76–79). Therefore, proliferation and SHM appear to occur in sequential and mutually exclusive cell states, DZp and DZd respectively, that ensure cells have exited cell cycle before initiating SHM. In this way, the attendant risks of gene mutation are mitigated.

B cells undergoing SHM-associated DNA damage and repair undergo an additional checkpoint prior to transit to the LZ. Upon completion of SHM, GC B cells replace old surface BCRs with the newly mutated BCR (80). B cells that generated nonfunctional BCRs due to SHM are fated for apoptosis prior to LZ entry. Thus, there are two levels of BCR selection per GC cycle, structural competency followed by relative affinity. We would postulate that those cells that express an incompetent BCR in the DZd might undergo retrograde GC cycling to be cleared by TBMs, the only known macrophage resident in the DZ (37, 66).

In our model, cells transit from the DZd to LZ without intervening proliferation. Therefore, only mutations that immediately arise on the DNA coding strand would be selected. Mutations on the non-coding strand must also be selected. However for these mutations to be selected, we predict that must arise during the preceding GC cycle and previous transit through the DZd. This would allow these single stranded mutations to be “fixed” by DNA replication in the DZp and therefore become “visible” for subsequent selection in the LZ. Alternatively, it is possible that a minor fraction of DZd cells migrate back to the DZp for proliferation and fixing of non-coding strand mutations.

### Selection in the LZ Also Determines Differentiation to PC and MB Cell Fates

In addition to cyclic re-entry into the DZ, LZ B cell interactions with T_FH_ govern GC exit into the PB or MBC fates (18, 81–83). While the LZ population that induces Myc contains cells destined for cyclic re-entry, this B cell pool also contains PB and MBC precursors (45, 84). PB precursors are BCL6^lo^CD69^hi^IRF4^+^ and express high-affinity BCRs (38, 45, 85). Commitment to the PC fate is associated with stable B:T_FH_ cell conjugates suggesting strong T cell help instructs PC differentiation (45).

In contrast MBCs develop from low-affinity B cell clones that receive low strength signals from T_FH_ (5, 86–88). Weak T cell help also results in low Myc and mTORC1 activation, which also predisposes to differentiation into MBCs (44). Overall, these studies indicate that high affinity BCRs and strong T_FH_ interactions predispose to PC differentiation while low affinity BCRs, and poor T cell help, leads to differentiation into MBCs.

## Conclusion

Here we provide a three-compartment model that segregates each fundamental GC B cell function, selection, proliferation and SHM, into distinct, separate cell states. This both mitigates the risk of SHM and allows coordination of molecular processes specific to each function. Furthermore, analysis of these three populations reveal just how molecularly dynamic the GC subsets are with large differences in genomic accessibility, transcription, protein expression and protein phosphorylation across each cell state. Therefore, it is likely that many regulatory mechanisms vertically integrate across the biosynthetic pathway to both drive and maintain the integrity of the GC response. Understanding these mechanisms, and how they integrate to regulate GCs, will provide opportunities to better treat a wide breadth of infectious, autoimmune and neoplastic diseases.

## Author Contributions

DK and MC wrote and edited the manuscript. All authors contributed to the article and approved the submitted version.

## Funding

This work is supported by the US National Institutes of Health, National Institute of Allergy and Infectious Diseases grant R01AI143778.

## Conflict of Interest

The authors declare that the research was conducted in the absence of any commercial or financial relationships that could be construed as a potential conflict of interest.
